# Frequency and Association Of GSTM1 and GSTT1 Gene Polymorphisms with Survival in Breast Cancer Patients

**DOI:** 10.31557/APJCP.2020.21.8.2251

**Published:** 2020-08

**Authors:** Phakarat Tangkheunkan, Kitiphong Harncharoen, Somchai Thanasitthichai, Danai Tiwawech, Wichai Purisa, Pensri Saelee, Ruxjinda Wattanalai

**Affiliations:** 1 *Faculty of Public Health, Mahidol University, Bangkok, Thailand. *; 2 *Research Division, National Cancer Institute, Bangkok, Thailand. *; 3 *Faculty of Pharmacy, Naresuan University, Phitsanulok, Thailand. *; 4 *Siam University, Bangkok, Thailand. *

**Keywords:** GSTM1, GSTT1, polymorphism, survival, breast cancer

## Abstract

**Objective:**

Glutathione S-transferase M1 and T1 (GSTM1 and GSTT1) are the key detoxification enzymes of xenobiotics, including chemotherapeutic drugs. The deletion polymorphisms of GSTM1 and GSTT1 genes are associated with reduced enzyme activity that influenced clinical outcomes of chemotherapeutic agents in breast cancer. However, there is limited information among Thai patients. This research aims to explore the frequency and role of GSTM1 and GSTT1 polymorphisms on survival among Thai patients with breast cancer.

**Methods:**

The retrospective cohort study was performed. Demographic data and clinicopathology characteristics were collected from hospital base registry data and medical records. A multiplex qualitative real-time PCR method was used to detect the presence or absence of the GSTM1 and GSTT1 gene in the genomic DNA samples of the participants.

**Results:**

The frequencies of the GSTM1 and GSTT1 null genotypes in 198 breast cancer patients were 65.70% and 33.30%, respectively. The overall survival at 1, 3 and 5 years were 95.00%, 83.00%, 71.00% respectively. The log rank test and Cox proportional hazards revealed a significant different in the 5-years overall survival according to lymph node metastasis and tumor stage (P = 0.014 and P < 0.001). No associations between overall survival and GSTM1 or GSTT1 genotype were found in single or combined genotypes analyses (P = 0.76 and P= 0.15).

**Conclusion:**

The results of our study provided the epidemiological information for prognostic of survival in breast cancer patients treated with chemotherapy.

## Introduction

Breast cancer is the common cancer and one of the leading causes of death among women worldwide, including Thailand. In 2018, the GLOBOCAN project reported that there were 2,088,849 new cases and 626,679 deaths of breast cancer among women worldwide (Bray et al., 2018). In Thailand, according to the National Cancer Institute (NCI)’ s report, breast cancer was the most leading new cancer among Thai women follow by cervix uteri cancer in 2015 (Imsamran et al.,2015).

As we know glutathione S-transferase M1 and T1 are the enzymes in a supergene family that involved in phase II biotransformation and play a key role in the detoxification of a broad range of xenobiotics. GSTM1 and GSTT1 enzymes catalyze the conjugation of glutathione to electrophilic compounds, resulting in less reactive and more immediately for excretion. Substrates of GST-catalyzed reactions include pre-carcinogens, such as polycyclic aromatic hydrocarbons, pharmacological drugs such as paracetamol, chemotherapeutic agents and free radicals generated during oxidative stress (Sreenivasulu et al., 2017). The deletion polymorphisms of *GSTM1* and *GSTT1 *genes are associated with reduced enzyme activity that could be associated with the susceptibility to breast cancer and treatment outcome (Zhang et al., 2017).

Because of the limited information regarding GSTM1 and GSTT1 polymorphisms among Thai patients with breast cancer, therefore, the objective of this research is to explore the frequency and association between these two genetic polymorphisms on survival in Thai patients with breast cancer. 

## Materials and Methods

*Study Design and study population*

The retrospective study was conducted based on data collected from breast cancer patients who were admitted and registered in the NCI, Thailand during January 1st, 2010 to December 31st, 2011. The total participants recruited in this study are 198 patients with newly diagnosis of breast cancer and histologically confirmed. All the participants were treated with chemotherapy.

*Study Procedure*

The chemotherapy includes anthracycline-based chemotherapy that consist of doxorubicin and cyclophosphamide. 

*Ethics Approval and Consent to Participate*

This study was approved by the Ethical Review Committee of Human Research, Faculty of Public Health, Mahidol University (protocol number 179/2560) and the Research Committee of NCI (project number 195_2017T_OUT525). 

*Data Collection*

Secondary data was collected. Demographic data and clinicopathology characteristics such as tumor size, tumor grade, tumor stage classified by TNM staging system according to The American Joint Committee on Cancer (AJCC), HER2 receptor, Ki-67 status, p53 status and hormone receptors were collected from hospital base registry data and medical records. 

*Data Analysis*

Descriptive statistics of patients were presented as mean and standard deviations for continuous measures, whereas frequencies were used for categorical measures. The power of the study is set at 80%. The statistical signiﬁcance of differences in genotype frequencies between participants were estimated by the Chi-square (*X*^2^) test. Binary logistic regression was used for all analysis variables to estimate risk as odds ratios (ORs) with 95 % conﬁdence intervals (95 % CIs). ORs were adjusted for confounding variables like age, tumor stage, tumor grade, hormone receptor and HER2 status. In calculating survival, cumulative 5-years survival rates were calculated starting from the date of diagnosis. Survival time was determined from cancer diagnosis to the end of follow-up, with vital status of alive or dead. Cases whose vital status was unknown at 5 years after diagnosis was assumed to be alive as of the last known date of living. Survival estimated was determined by Kaplan Meier method and differences in survival was compared by the log-rank test. A Cox regression model was used to calculate the hazards ratio of death, take into account the genetic polymorphisms of GSTM1, and GSTT1 and other factors. All the tests were set at significance level of 95%. All statistical analysis was performed using SPSS version 10 (2007).

*Genotyping Protocol*

The extracted DNA from buffy coat collected from 198 breast cancer patients were kept at -80^o^C prior to analysis. A multiplex qualitative real-time PCR method was used to detect the presence or absence of the *GSTM1* and *GSTT1* gene in the genomic DNA samples of the participants. The assay was performed in the StepOnePlus Real-Time PCR System (Applied Biosystems, U.S.A.). The Express SYBR Green qPCR Super Mix Universal (Invitrogen, U.S.A.) was used as the master mix. All primers were ordered from Macrogen, Korea. Determination of the null *GSTM1* polymorphism was performed using the following primers 5’- GAA CTC CCT GAA AAG CTA AAG C-3’ and 5’- GTT GGG CTC AAA TAT ACG GTG G-3’, for GSTT1, using primers 5’-TCT CCT TAC TGG TCC TCA CAT CTC-3’ and 5’-TCA CCG GAT CAT GGC CAG CA-3’. The internal control was performed by the human β-globin using primers 5’-AAC TTC ATC CAC GTT CAC C-3’ and 5’-GAA GAG CCA AGG ACA GGT AC-3’. The protocol was slightly modified from previous study (Tiwawech et al., 2011).

## Results

*The distribution for general and clinicopathological characteristics*

Total participants in this study included 198 patients with primary invasive ductal carcinoma breast cancer who had admitted at NCI. According to the general and clinicopathological characteristics of participants at diagnosis, there was 57.00% of participants who were above 50 years old with a median age of 50.50 years. With regards to the tumor size, 43.43% of participants had a tumor with diameter 2.50 cm or less, while 56.57% of participants had a tumor size larger than 2.50 cm. The result showed that 55.05% of participants had lymph node metastasis. As regard to the tumor grade, there are 6.06%, 48.48% and 45.46% of participants who had tumor grade I, II, and III, respectively. For tumor stage according to AJCC, mostly of participants were in stage II (56.57%) and stage III (34.34%). About hormone receptors status, 61.11% and 47.47% of participants had estrogen receptor positive and progesterone receptor positive, respectively. Consider with HER-2 receptor status, there are 15.66% of participants showed positive result. For the proliferation marker Ki-67 status and tumor suppressor p53 status, the frequencies of Ki-67 positive and p53 positive were 74.24% and 68.69% respectively. 

*The frequency of GSTM1 and GSTT1 gene polymorphisms*

Among breast cancer patients, genotypes and allele distributions of GSTM1 and GSTT1 gene polymorphisms are summarized in Table 1. The GSTM1 and GSTT1 null genotype frequencies were 65.70% and 33.30%, respectively. Whereas, the frequency of combine GSTM1 and GSTT1 null genotype was 21.20%.

*Association of overall survival with clinicopathological characteristics among primary invasive ductal carcinoma*

**Figure 1:**
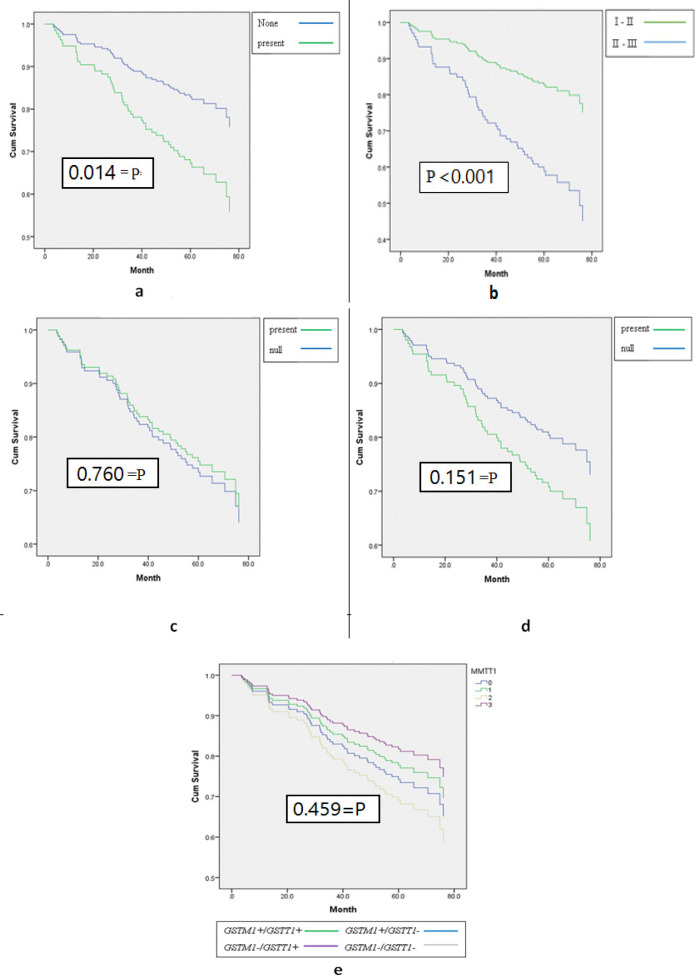
a, overall survival by lymph node metastasis; b, overall survival by tumor stage; c, overall survival by GSTM1 gene polymorphism; d, overall survival by GSTT1 gene polymorphism; e, overall survival by GSTM1 and GSTT1 gene combined polymorphisms

Associations between overall survival and prognosis clinicopathological characteristics was showed in Table 2. Among total, 57 deaths were determined in this study participants of 198 breast cancer patients. The probability of overall survival at 1, 3 and 5 years was 0.95, 0.83 and 0.71, respectively. After analyzing by the log rank test and Cox’s proportional hazards model, the results showed a significant different in the probability of overall survival at 5 years according to lymph node status and stage of tumor (P = 0.014 and P < 0.001) and the Kaplan-Maier curves as show in Figure 1. 

Patients without lymph node involvement had better 5 years overall survival probability than patients with lymph node involvement (P = 0.014). The probability of survival at 5 years among patients without lymph node involvement and patients with lymph node status were 0.83 and 0.67, respectively. Patients with lymph node involvement had around 2-fold-higher risk of death compared with patients without lymph node involvement (HR = 2.105, 95% CI = 1.148 - 3.859, P = 0.016).

According to early-stage tumor (stage I and II) patients had greater overall survival probability at 5 years than patients with advanced-stage tumor (stage III and stage IV) (P < 0.001). The probability of survival at 5 years among patients with early-stage tumor and patients with advanced-stage tumor were 0.80 and 0.56, respectively. Patients with advanced-stage tumor had nearly 3-fold-increased risk of death compared with patients with early-stage tumor (HR = 2.782, 95% CI = 1.587 - 4.875, P < 0.001). Furthermore, no significant association between survival probability at 5 years and other characteristics.

*The association between survival and GSTM1 and GSTT1 gene polymorphisms*

In table 3, there was no relations of overall survival and GSTM1 or GSTT1 genotype in single genotype or combined genotypes analyses. 

The probability of survival at 5 years among GSTM1 null genotype and present genotype were 0.71 and 0.73 respectively (P value = 0.760). Likewise, comparison of patients harboring GSTM1 null genotype and patients harboring GSTM1 present genotype had same risk of death (HR = 1.097, 95% CI = 0.605 - 1.987, P = 0.761). 

Regard with GSTT1 gene polymorphisms, the probability of survival at 5 years among GSTT1 null genotype and GSTT1 present genotype were 0.78 and 0.68 respectively (P = 0.151). Patients harboring GSTT1 null genotype had a lower risk of death compared with patients harboring GSTT1 present genotype (HR = 0.632, 95% CI = 0.336 - 1.189, P = 0.154).

With respect to combined genotypes analyses, the hazard ratios among patients with GSTM1+/GSTT1+, GSTM1+ /GSTT1-, GSTM1-/GSTT1+ compared with GSTM1-/GSTT1- were 1.481, 1.250 and 1.838 respectively nevertheless, there was not statistically signiﬁcant difference (P = 0.398, 0.696, 0.132). 

Moreover, in this study, the Cox’s proportional hazard ratio was analyzed and calculated for adjusted hazard ratios. After adjusted for tumor grade and progesterone receptor status, patients harboring GSTT1 null genotype had a lower risk of death compared with patients with GSTT1 present genotype (HR = 0.630 and 0.612) nevertheless, there was not statistically signiﬁcant difference (P = 0.152 and 0.143). With respect to combined genotypes analyses, after adjusted for progesterone receptor status, the hazard ratios among patients with GSTM1+/GSTT1+, GSTM1+ /GSTT1-, GSTM1-/GSTT1+ compared with GSTM1-/GSTT1- were 1.837, 1.653 and 2.018 respectively nevertheless, there was not statistically signiﬁcant difference (P = 0.209, 0.393, 0.104).

**Table 1:** Genotype and Allele Frequencies of GSTM1 and GSTT1 gene Polymorphisms in Patients with Breast Cancer (N = 198)

Gene	Frequencies	N (%)
GSTM1	Present	68 (34.30)
	Null	130 (65.70)
GSTT1	Present	132 (66.70)
	Null	66 (33.30)
GSTM1 and GSTT1 combined	GSTM1+/GSTT1+	44 (22.20)
	GSTM1+/GSTT1-	24 (12.10)
	GSTM1-/GSTT1+	88 (44.40)
	GSTM1-/GSTT1-	42 (21.20)

**Table 2:** Overall Survival Probability at 5 Years by Clinicopathological Characteristics in Breast Cancer Patients

Characteristics	No. of patients	No. of deaths	Probability of survival at 5 years	P value^a^	Unadjusted HR (95% CI)
All patients	198	57	0.71	-	
Age					
< 50 years	74	21	0.72		Reference
≥ 50 years	101	29	0.75	0.83	1.064 (0.606-1.865)
Tumor size					
≤ 2.50 cm	72	15	0.79		Reference
>2.50 cm	103	35	0.66	0.142	1.769 (0.966-3.240)
LN metastasis					
None	77	15	0.83		Reference
Present	98	35	0.67	0.014*	2.105 (1.148-3.859)
Tumor grade					
I	10	1	0.9		Reference
II - III	161	49	0.72	0.17	3.651 (0.504-26.450)
Tumor stage					
I - II	112	22	0.8		Reference
III - IV	63	28	0.56	<0.001*	2.782 (1.587-4.875)
Estrogen receptor					
Positive	111	28	0.75		Reference
Negative	60	21	0.65	0.123	1.558 (0.883-2.746)
Progesterone receptor					
Positive	85	23	0.73		Reference
Negative	80	24	0.7	0.561	1.185 (0.668-2.103)
HER-2 receptor					
Positive	28	10	0.64		1.456 (0.725-2.925)
Negative	142	38	0.76	0.288	Reference
Ki-67					
Positive	130	37	0.72		1.319 (0.518-3.358)
Negative	23	5	0.78	0.56	Reference
p53					
Positive	119	30	0.75		Reference
Negative	24	11	0.54	0.052	1.960 (0.981-3.913)

*, significantly different; ^a^, P values from the log rank test

**Table 3:** Overall Survival at 5 Years by GSTM1 and GSTT1 gene Polymorphisms in Breast Cancer Patients

Genotype	No. patients	No. deaths	5 years survival probability	*P*- value^a^	Unadjusted HR (95% CI)	*P*-value^b^
GSTM1						
Present	59	16	0.73		Reference	
Null	116	34	0.71	0.76	1.097 (0.605-1.987)	0.761
GSTT1						
Present	116	37	0.68		Reference	
Null	59	13	0.78	0.151	0.632 (0.336-1.189)	0.154
GSTM1 and GSTT1 combined						
GSTM1-/GSTT1-	39	8	0.81		Reference	
GSTM1+/GSTT1+	39	11	0.72		1.481 (0.595-3.681)	0.398
GSTM1+/GSTT1-	20	5	0.8	0.459	1.250 (0.409-3.822)	0.696
GSTM1-/GSTT1+	77	26	0.69		1.838 (0.832-4.063)	0.132

^a^, P values from the log rank test; ^b^, P value of unadjusted HR

## Discussion

In this study, the overall survival at 1, 3 and 5 years was 95.00%, 83.00% and 71.00%, respectively. There were some studies conducted in Thailand to determine the survival of breast cancer patients. In 1995, Sriamporn et al., (1995) indicated the 5 years survival of breast cancer from the population-based study in Khon Kaen province registered in the period 1985-1992 was 48.10%. Then in 2000, Poum et al., (2012) conducted the study at a teaching university in northeast of Thailand among 340 female breast cancer patients and followed-up until the end of 2006 and the 5 years survival was 42.90%. Moreover, Kongsiang et al., (2014) conducted the study in 1999-2009 to evaluate the relation of molecular subtypes and survival among breast cancer patients treated with radiotherapy and the 5 years overall survival was 59.20%. The difference of those outcomes may cause by study design, the patient characteristics and study period.

This could be explained that nowadays breast cancer patients trended to improve the survival due to the early screening policy and/or the current standard treatment that allowed the effective treatment outcome and low toxicity (Cedolini et al., 2014; Furrukh et al., 2018). 

The survival of breast cancer patients varied among countries. The survival of breast cancer patients in developed countries is higher than we found in this study. The SEER 5-year survival rate in United States (2010-2016) was 90.00% in females and 83.60% in males (Howlader et al., 2019). The study in Europe 28 countries reported the survival rate at 5 years ranges from 79% to 93% (Allemani et al., 2018). Data collected in developing countries showed either the same or vary of the 5 years survival (Shulman et al., 2010). The international diversity of survival in breast cancer was not easy to interpret may be due to many factors such as the knowledge and awareness of patients, the early detection, availability of effective treatment and health services accessibility which may be different between developed countries and developing countries. 

A key prognostic parameter of patients with breast cancer survival is stage at diagnosis (Zuo et al., 2017). Breast cancer patients with early-stage showed much higher survival rates than those with late-stage. Several studies showed that survival significantly diverged to stage at diagnosis. As Walters et al., (2013) investigated the differences in breast cancer at 3 year survival in developed countries including Canada, Denmark, Norway, Sweden, and the United Kingdom according to the stage at diagnosis. The result showed that approximately 30.10% - 45.20% of patients were diagnosed with tumor stage I, 39.0% - 47.70% with tumor stage II, 3.50% - 15.30% with tumor stage III, and 2.90% - 6.90% with tumor stage IV. In our study population revealed a greater frequency of stage II (56.57%) and stage III (34.34%) compared with those in developed countries which may emphasize the importance for screening program for primary prevention. 

Conform to several studies that focused on lymph node metastasis status as the predictive factors for breast cancer survival thus the lymph node metastasis is linked to distant recurrence and survival of patients (Yang et al., 2017; Blenman et al., 2018; Mahmood et al., 2015). Respect to lymph node status, patients without lymph node metastasis had much greater survival rates than those with lymph node metastasis. In this study, lymph node metastasis was analyzed as the dichotomous variables; none and present, in contrast to some studies analyzed as the number of nodes involved or lymph node ratio (proportion of number of lymph nodes that are positive metastasis to the total number of lymph nodes evaluated). For instances, Mahmood et al., (2015) observed that patients with less than 5 nodes metastasis survived for more than 10 years were 16.50% compare with patients with more than 9 nodes metastasis survived were only 5%. Therefore, it can imply that survival was decreased while number of nodes metastasis was increased. Hung et al., (2018) revealed that lymph node ratio was a proper prognosis factor of survival than TNM system from the American Joint Committee on Cancer (AJCC).

Breast cancer is the global public health problem including Thailand. The information about genetic variation in breast cancer in Thailand may be limited. In this study, 198 breast cancer patients were genotyped, and the result showed that among Thai breast cancer patients, the frequency of the GSTM1 and GSTT1 null genotype was 65.70% and 33.30%, respectively. Several studies reported the frequency of the GSTM1 and GSTT1 null genotype in breast cancer patients. Nevertheless, Pongtheerat et al., (2009) showed the frequencies of the GSTM1 and GSTT1 null genotype in Thai patients with breast cancer was (14/40) 35.00% and (18/43) 41.90%, respectively. The findings from our study showed the greater frequency of GSTM1 null genotype (65.70%) in breast cancer patients than the finding from the former study (35.00%).

Several evidences showed that the genetic polymorphisms of drug transporters, drug-metabolizing enzymes and drug targets are involved in inter-individual diversity of the efficacy and toxicity of chemotherapy and several medicines (Tecza et al., 2016; Chen et al., 2018; Li et al., 2018). A personalized chemotherapy is proposed to be a promising tool to increase chemotherapy response, prevent the toxicity and elevate overall survival of patients with breast cancer. As we know that the GST super-family belongs to the phase II biotransformation enzymes, which function a crucial part in the biotransformation or detoxification of a variety of xenobiotics as well as chemotherapeutic agents. However, there were several studies focused on the role of GSTs in chemotherapy efficacy and treatment outcome, the results of those studies have indicated the inconsistent association (Egan et al., 2004; Abbas et al., 2015; Huang et al., 2003; Lizard-nacol., 1999; Yang et al., 2005). 

Indeed, our results could not find the relation of GSTM1 and GSTT1 gene polymorphisms and the overall survival among patients with breast cancer treated with chemotherapy. Conform to the result from Lizard-nacol et al., (1999) which found that GSTM1 gene polymorphism did not associate with clinicopathology characteristics, clinical outcomes of chemotherapeutic agents in advanced breast cancer. Moreover, Yang et al., (2005) indicated that there was no relation to any of the GSTM1 or GSTT1 polymorphisms as potential role in prognosis to the clinical outcomes and overall survival of breast cancer patients after chemotherapy. On the other hand, some studies indicated the contrast results, as Wang et al., (2015) reported that the GSTM1 null genotype was related with a greater to chemotherapeutic agents response and the odds ratio was 1.78 (95% CI = 1.03 - 3.08) and the hazard ratio for overall survival in patients with the GSTM1 null genotype was 0.57 (95% CI = 0.32 - 0.98) compare with GSTM1 present genotype. However, they indicated that there was no statistically significant relation of the GSTT1 polymorphisms and overall survival among breast cancer patients. Another study from China, Liu et al., (2014) found that patients with GSTM1 null genotype related to worse overall survival of breast cancer patients treated with chemotherapy and the hazard ratio for overall survival was 2.00 (95% CI = 1.15 - 3.48). Furthermore, Bai et al., (2012) evaluated the prognostic role of GST gene polymorphisms among patients with breast cancer treated with neoadjuvant chemotherapy and the result showed that patients with the GSTM1 null genotype had a better survival and statistical significantly lower risk of death than patients harboring GSTM1 present genotype (HR = 0.66, 95% CI = 0.31- 0.93). These differences results may be cause of methodology, study design, study population and sample size, genotyping methods or chemotherapy regimens. In conclusion, this study is the first study to explore the role of GSTM1 and GSTT1 polymorphisms with survival in breast cancer patients in Thailand.
